# Internalized weight stigma and intuitive eating among stressed adults during a mindful yoga intervention: associations with changes in mindfulness and self-compassion

**DOI:** 10.1080/21642850.2021.1992282

**Published:** 2021-11-19

**Authors:** Tosca D. Braun, Kristen E. Riley, Zachary J. Kunicki, Lucy Finkelstein-Fox, Lisa A. Conboy, Crystal L. Park, Elizabeth Schifano, Ana M. Abrantes, Sara W. Lazar

**Affiliations:** a Department of Psychiatry and Human Behavior, Alpert Medical School of Medicine, Brown University, Providence, RI, USA; b Centers for Behavioral and Preventive Medicine, The Miriam Hospital, Providence, RI, USA; c Department of Psychological Sciences, University of Connecticut, Storrs, CT, USA; d Department of Clinical Psychology, Graduate School of Applied and Professional Psychology, Rutgers University, New Brunswick, NJ, USA; e Department of Psychiatry, Massachusetts General Hospital/Harvard Medical School, USA; f Beth Israel Deaconess Medical Center, Harvard Medical School, Boston, MA, USA; g Department of Statistics, University of Connecticut, Storrs, CT, USA; h Behavioral Medicine and Addictions Research, Butler Hospital, Providence, RI, USA; i Department of Psychiatry, Massachusetts General Hospital, Boston, MA, USA; j Department of Psychology, Harvard Medical School, Boston, MA, USA

**Keywords:** Yoga, intuitive eating, internalized weight bias or internalized weight stigma, mindfulness, self-compassion

## Abstract

**Purpose:**

Internalized weight stigma (IWS) is common in the United States of America across body weight categories, and is implicated in the development of distress and unhealthy eating behaviors (e.g. overeating, disordered eating) that can foster poor cardiometabolic health. While emerging intervention research shows early promise in reducing IWS, long-term efficacy is unclear and novel strategies remain needed. This analysis examined whether participation in a mindful yoga intervention was associated with reduced IWS and increased intuitive eating, an adaptive eating behavior, and whether these changes correlated with each other or with changes in mindfulness and self-compassion.

**Methods:**

Participants were stressed adults with low fruit and vegetable intake (*N *= 78, 64.1% White, M. Body Mass Index  25.59 ± 4.45) enrolled in a parent clinical trial of a 12-week mindful yoga intervention. Validated self-report measures of IWS, intuitive eating, mindfulness, and self-compassion were administered at pre-treatment, mid-treatment (8 weeks), post-treatment (12 weeks), and 4-month follow-up (24 weeks).

**Results:**

Linear mixed modeling revealed significant improvements in IWS and intuitive eating across the four timepoints (*p* < .001). Reduced IWS correlated with increased intuitive eating pre- to post-treatment (*p *= .01). Improved self-compassion and mindfulness correlated with intuitive eating (both *p *= . 04), but not IWS (*p *= .74 and *p *= .56, respectively*)*.

**Conclusion:**

This study offers preliminary support for the hypothesis that mindful yoga may promote intuitive eating and reduce IWS among stressed adults with poor diet, and suggests that changes in these factors may co-occur over time. Further investigation with controlled designs is necessary to better understand the temporality and causality of these relationships.

**Trial registration:** ClinicalTrials.gov identifier: NCT02098018.

A recent international consensus statement expressed the alarming public health consequences of social stigma directed towards people with obesity (i.e., weight stigma) ) and its sequelae, meriting action and further research towards its mitigation (Rubino et al., 2020). Internalized weight stigma (IWS) refers to the application of weight-related stereotypes towards oneself (i.e. self-stigma). Common across weight statuses, IWS is implicated as a dual risk factor for eating pathology and weight gain, given its associations with binge eating, emotional eating, and food addiction in diverse samples (Pearl & Puhl, 2018). Yet, research examining effective interventions to reduce IWS suggests limited efficacy (Pearl, Wadden, Bach, Tronieri, & Berkowitz, 2020), and little work has examined protective factors that may mitigate IWS’s adverse effects on behavioral health. Such protective factors include mindfulness, implicated protective against other forms of internalized stigma (e.g. mental illness self-stigma; Yang & Mak, 2016), self-compassion, suggested in early research to be depleted by and/or protective against the effects of IWS and related factors (Braun et al., 2021a; Webb & Hardin, 2015), and intuitive eating, referring to the recognition and honoring of endogenous hunger and satiety cues (Tylka, 2006).

Intuitive eating comprises three facets: (a) eating primarily for physical rather than emotional reasons, (b) unconditional permission to eat (as opposed to dieting or following specific dietary rules, which can lead to binging), and (c) reliance on internal hunger and satiety cues (Tylka, 2006). Intuitive eating training is commonly offered in eating pathology and non-dieting interventions (i.e. programs that promote behavioral health independent of a focus on dieting or body weight) (Clifford et al., 2015) to combat the effects of IWS and the dieting mindset on negative affect and overeating that can follow consumption of ‘bad’ or ‘forbidden’ foods (Mathieu, 2009). Growing, primarily observational research suggests intuitive eating may improve body image (Keirns & Hawkins, 2019) and offer protection against eating pathology, binge eating, and weight gain while promoting adaptive dietary choices (Bruce & Ricciardelli, 2016; Warren, Smith, & Ashwell, 2017).

Early research examining the links between IWS and intuitive eating suggests IWS may be a risk factor for less intuitive eating. IWS predicted fewer gains in intuitive eating among women in a healthy lifestyle program (Mensinger, Calogero, & Tylka, 2016), and in a cross-sectional study of college women, was associated with less intuitive eating through lower body image flexibility (i.e. accepting rather than avoiding negative body image-related cognitions and affects) and marginally lower self-compassion (i.e. treating oneself as a loved one might during pain or difficulty) (Webb & Hardin, 2015). This finding aligns with growing evidence that self-compassion is inversely associated with and potentially protective against the effects of IWS and related factors, and may promote healthy diet and eating behaviors such as intuitive eating (Biber & Ellis, 2017; Braun, Park, & Gorin, 2016a; Braun et al., 2021; Rahimi-Ardabili, Reynolds, & Vartanian, 2018; Wong, Knee, Neighbors, & Zvolensky, 2019). For instance, higher daily self-compassion was linked to improved intuitive eating and body image in a recent daily diary study (Kelly & Stephen, 2016). A self-compassionate response to emotional difficulty, including IWS-related distress, may improve attentiveness to and meeting of one’s emotional and physical needs and therefore, decrease IWS and/or intuitive eating.

A prerequisite to self-compassion is mindfulness – individuals must be *mindful* of distress before responding with self-compassion (Germer & Neff, 2019; Neff, 2003). Mindfulness has been consistently associated with healthier eating behaviors including intuitive eating, less eating pathology, and improved body image (Sairanen et al., 2015; Sala, Shankar Ram, Vanzhula, & Levinson, 2020; Warren et al., 2017). Mindfulness may also protect against the adverse effects of internalized stigma on psychological wellbeing (Chan & Leung, 2021; Yang & Mak, 2016). Further, intuitive eating training emphasizes the importance of both mindfulness and self-compassion (Tribole & Resch, 2012). Thus, improvements in mindfulness and self-compassion may correspond with those of intuitive eating and IWS. Yet to our knowledge, no prospective research has yet examined associations between changes in these constructs over time.

Mindful *hatha* yoga – a form that emphasizes compassionate mindfulness of present-moment experience (Cook-Cottone & Douglass, 2017) – represents an optimal context in which to examine prospective associations between changes in IWS and intuitive eating with those of mindfulness and self-compassion. Hatha yoga (henceforth referred to as yoga) is an integrative mind–body practice with roots in ancient India that primarily comprises breathing exercises, postures, meditation, and relaxation in present-day United States of America (U.S.A.) and other countries (Desikachar, 1999; NCCIH, 2020). Yoga that is independent of explicit dietary prescription or principles has been associated with healthy eating and weight maintenance (Kristal, Littman, Benitez, & White, 2005; Lauche, Langhorst, Lee, Dobos, & Cramer, 2015; Watts, Rydell, Eisenberg, Laska, & Neumark-Sztainer, 2018) and implicated as potentially effective in the complementary treatment of eating pathology (Borden & Cook-Cottone, 2020; Domingues & Carmo, 2019; Neumark-Sztainer, 2014).

Mindful yoga promotes positive embodiment (i.e. ‘the ability to sense and feel through the body in the present moment’) (Cook-Cottone & Douglass, 2017, p. 1) and the closely-associated factors of mindfulness and self-compassion (Braun et al., 2016b; Gaiswinkler & Unterrainer, 2016; Riley et al., 2016; Shelov, Suchday, & Friedberg, 2009). Cox and Tylka (2020) theorize that mindful yoga-related increases in mindfulness and self-compassion support the development of positive embodiment, including the embodied practice of intuitive eating. Further, improvement in eating pathology risk factors linked to IWS have been shown to improve during yoga, including self-objectification (i.e. the process whereby individuals internalize sexual objectification of their bodies and evaluate their body as an object that appears to others) (Fredrickson & Roberts, 1997) and poor body image (Borden & Cook-Cottone, 2020). Yet no research to our knowledge has examined whether yoga practice is associated with reduced IWS, and only one cross-sectional study has examined intuitive eating in any yoga-related context, finding that it positively associates with factors related to positive embodiment (i.e., body satisfaction, body awareness, and body responsiveness) in a sample of regular female yoga practitioners (Dittmann & Freedman, 2009).

As an initial step towards addressing these gaps, the present secondary analysis of a pilot clinical trial examined changes in IWS and intuitive eating during participation in a 12-week mindful yoga program among stressed adults with poor diet. We hypothesized participants would report decreased IWS (Hypothesis 1) and increased intuitive eating (Hypothesis 2) at 12 weeks (post-treatment), and that these changes would be maintained at 24 weeks (follow-up). We secondarily examined associations between changes in these constructs from pre- to post-treatment, and hypothesized that changes in IWS would be inversely correlated with those of intuitive eating (Hypothesis 3). As a tertiary inquiry, we examined whether changes in mindfulness or self-compassion (both reported in our prior work; Park, Finkelstein-Fox, Sacco, Braun, & Lazar, 2020) were associated with changes in intuitive eating (Hypothesis 4a and 4b, respectively) and IWS (Hypothesis 5a and 5b, respectively).

## Materials and methods

The results presented here comprise a secondary analysis of a parent study that assessed changes in fruit and vegetable intake (primary) and stress (secondary), reported elsewhere (Braun et al., 2021b; Park et al., 2020). An additional aim of the parent study was to examine the optimal dose of home yoga practice assignment on change in dietary behaviors and stress. In addition to the 12-week in-person yoga intervention delivered to all participants, participants were randomly assigned to receive one of three home yoga practice conditions: low practice (10 min. per day, six days per week), high practice (40 min. per day, six days per week), and hybrid practice (10 min. per day three days per week, and 40 min. per day three days per week). Detail on home practice adherence and frequency can be viewed in our published work (Greenberg et al., 2018). Because the present study observed no group differences on the variables of interest in this study, the reported analyses analyze all participants as one group.

### Participants

A total of 84 participants were enrolled, 78 of whom completed assessments for the measures of interest and were retained in analysis. Most enrolled were female (70.5%), non-Hispanic (87%), and 4-year college educated (85.9%). Reported racial identities included American Indian or Alaskan Native (1.3%), Asian (12.8%), Black or African American (2.6%), Multiracial (11.5%), White (64.1%), Other (3.8%), or prefer not to report (3.8%). Participants’ average Body Mass Index (BMI) was 25.59 (*SD *= 4.45) and most BMI categories were represented, ranging from 19.03 (‘Normal’) to 39.9 (‘Class II obesity’), with an average age of 39.42 (*SD* = 14.16).

Healthy, yoga-naïve participants self-identified as stressed were recruited from two sites and their surrounding communities in the Northeastern U.S. – a rural public university and an urban academic medical center. Inclusion criteria included being 23–67 years of age and self-report of feeling both ‘stressed’ (yes/no checkbox) and consuming 5 or fewer servings of fruits and vegetables/day (assessed through research assistant query, with validated serving size prompts) (NIH National Heart Lung and Blood Institute, 2013; Paxton, Strycker, Toobert, Ammerman, & Glasgow, 2011). Exclusion criteria included factors that may impact diet or body weight, including currently trying to lose weight, an exercise regimen of more than 180 min per week (based on Haskell et al., 2007), current diagnosis of psychiatric illness or prior eating disorder diagnosis as determined by the MINI or SCID eating disorders module, significant prior meditation or yoga experience (defined as ≥12 classes in last 3 years or more than 20 classes in lifetime), and medications that altered appetite. Medical conditions that would limit the ability to exercise or do yoga, including BMI > 40, were also excluded.

### Procedures

Following initial baseline assessment of eligibility (T1), this study included three additional assessment points at 8 weeks (T2; mid-treatment), 12 weeks (T3; post-treatment), and 24 weeks (T4; follow-up). Participants were remunerated up to $100 for completing study assessments ($25 per time-point) and received the yoga program for free. The study protocol was approved by the Institutional Review Boards (IRBs) of University of Connecticut and Massachusetts General Hospital, and monitored by Westat. The protocol is registered in Clinicaltrials.gov (*#*NCT02098018).

### Yoga intervention

The Kripalu yoga-based stress management intervention was designed to integrate yoga practice with elements of yoga philosophy pertinent to self- and emotion-regulation (e.g. mindfulness, self-compassion) to decrease physiological arousal and enhance well-being. Details on the intervention, including the three home practice conditions, can be reviewed in our prior work (Braun et al., 2021; Greenberg et al., 2018). Broadly, the 12-week intervention comprised two segments: first, a manualized protocol consisting of eight weekly 2-hour sessions on “hatha” (since technically the entire intervention, including philosophy components, were yoga), whereas the 90-min classes were hatha yoga practices only. The initial eight-week protocol was created and piloted with the Kripalu Center for Yoga and Health and was adapted for use with this population by the first author, a certified yoga therapist, with an experienced registered yoga instructor with curriculum expertise. The intervention avoided any mention of diet, weight stigma, body image, eating behaviors, or weight loss/maintenance to avoid confounding the primary outcome metric in the parent study (i.e. non-prescribed changes in fruit and vegetable intake).

### Measures

All measures were assessed at baseline (T1), mid-program (T2), 12 weeks (T3; post-treatment), and 24 weeks (T4; follow-up).

*Internalized weight stigma (IWS)* was assessed with the 11-item Weight Bias Internalization Scale-Modified (WBIS-M) (Pearl & Puhl, 2014), a modification of the original WBIS for use with persons of all body weights. Items are ranked on a 7-point Likert scale ranging from *strongly disagree* (1) to *strongly agree* (7); higher scores indicate greater internalized weight stigma. The measure produces a global score of internalized weight bias (Durso, Latner, & Ciao, 2016), conceptually paralleling the construct of internalized weight stigma. The WBIS-M has demonstrated adequate internal consistency and predictive validity. In the present study at T1, alpha was 0.90.

*Intuitive eating* was assessed with the 23-item Intuitive Eating Scale-2 (IES-2) (Tylka, Van Diest, & M, 2013), a revision of the original 21-item IES (Tylka, 2006). Items are ranked on a 5-point Likert scale ranging from *strongly disagree* (1) to *strongly agree* (5); higher scores indicate higher intuitive eating. The IES-2 produces subscales and an overall intuitive eating score, the latter reported here. Prior research has indicated strong internal consistency reliability and validity of the IES-2 (Tylka, 2006; Tylka, Van Dienst, & M, 2015). In the present study at T1, alpha was 0.90.

*Mindfulness* was measured in this secondary analysis using the Acting With Awareness (AWA) subscale of the 24-item Five-Facet Mindfulness Questionnaire, short form (FFMQ-SF) (Bohlmeijer, ten Klooster, Fledderus, Veehof, & Baer, 2011), a brief version of the original 39-item FFMQ (Baer, Smith, Hopkins, Krietemeyer, & Toney, 2006). The AWA subscale was selected due to its strong association with the Mindful Attention Awareness Scale, a unidimensional measure of mindfulness (Baer et al., 2006; Brown & Ryan, 2003). Item responses range from *never or very rarely true* (1) to *very often or always true* (5); higher scores delineate greater mindfulness. The AWA subscale has demonstrated good reliability and validity (Bohlmeijer et al., 2011). Within this sample at T1, alpha was 0.82.

*Self-compassion* was assessed with the 12-item Self-Compassion Scale, Short-Form (SCS-SF) (Raes et al., 2011), a brief version of the original 26-item SCS (Neff, 2003). Item responses range from *almost never* (1) to *almost always* (5); higher scores denote greater self-compassion. The SCS-SF yields six subscales (self-kindness, self-judgment, common humanity, isolation, mindfulness, over-identification) and a global score, the latter used in the present analysis. Good internal consistency was shown for the SCS-SF global score in the validation study (Raes et al., 2011). Within this sample at T1, alpha was 0.88.

### Data analyses

The parent study was conservatively powered (*N* = 134, accounting for 20% drop-out), based on preliminary data (Salmoirago-Blotcher, Morgan, Fischer, & Carmody, 2013). Our enrollment volume fell short and the study was underpowered (*N* = 77 across both sites with baseline survey data in the present analysis). Normality of numerical study variables was examined, followed by computation of scale reliabilities, descriptive statistics, and bivariate correlations. Independent t-tests and Pearson’s chi-square tests were used to assess baseline differences between intervention completers vs. non-completers and assessment completers vs. non-completers. For these preliminary analyses, an alpha level (type 1 error rate) of .05 and complete case analysis were used.

Appropriate analyses for Hypotheses 1 and 2 – examination of change in internalized weight stigma (IWS) and intuitive eating over time – was determined via graphical analysis of distributions across time-points. To account for within-subject correlation and provide estimates for changes over time, longitudinal analyses used random intercept linear mixed-effects models (LMM) and a compound symmetry covariance structure in SPSS 25.0. This method uses maximum likelihood methods of parameter estimation and does not require complete cases (Jennrich & Schluchter, 1986). Time was examined as a categorical predictor. Where effects by time were significant, to determine whether the yoga intervention was associated with improved outcomes within the LMM, baseline scores (T1) were contrasted with those at 12 weeks (T3), with additional changes at 24 weeks (T4) assessed by contrasting T3 and T4 scores. The Bonferroni adjustment for multiple comparisons was applied to these analyses (*p*<.03). Analyses covary for sex and site because the randomization procedure in the parent study stratified participants by these variables, as well as age, given evidence that IWS and dietary factors related to intuitive eating may differ by age (Marvin-Dowle, Kilner, Burley, & Soltani, 2018; Pearl & Puhl, 2018). Because analyses computed with and without outliers yielded similar results, outliers were included in analyses.

To test associations between changes in intuitive eating and IWS (Hypothesis 3) as well as correlations of these factors with mindfulness (Hypothesis 4a, b) and self-compassion (Hypothesis 5a, b) from pre- (T1) to post-treatment (T3), we first generated standardized residualized change scores by regressing post-treatment T3 scores on T1 scores for each construct, controlling for sex, site, and age. We then examined whether changes in intuitive eating and IWS correlated with each other and mindfulness or self-compassion by computing Pearson’s correlations on these residualized change scores. For correlation analyses, alpha level was set at *p*<.05.

## Ethics statement

The study was approved by the Institutional Review Boards of the Massachusetts General Hospital (#2013P001153) and the University of Connecticut (#H14-215).

## Results

### Participants

**Figure 1.**
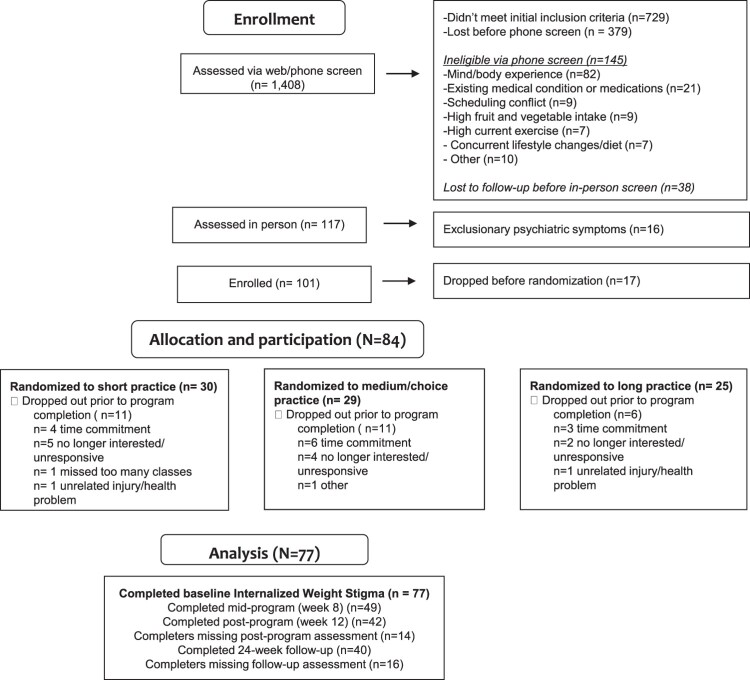
CONSORT diagram.

No differences in pre-treatment constructs were observed between assessment completers and non-completers at any timepoint (*p* > .05). Sixty-seven percent of study participants (*n*=56) completed the intervention (i.e. attended 5 or more classes), of whom 42 completed post-treatment and 40 completed follow-up assessments for IWS. Information on study attrition and completion rates are detailed in the CONSORT diagram, Figure 1. Descriptive statistics for constructs at each time-point are displayed in Table 1.

**Table 1.** Means and standard deviations for outcome variables at each time-point.

Outcome	T1		T2		T3		T4	
	*M (SD)*	*n*	*M (SD)*	*n*	*M (SD)*	*n*	*M (SD)*	*n*
IWS	2.66 (1.31)	77	2.39 (1.33)	48	2.27 (0.86)	41	1.98 (1.01)**	39
Intuitive Eating	3.37 (0.63)	75	3.49 (0.60)	48	3.57 (0.66)*	40	3.67 (0.70)*	29

*p ≤ .025, **p ≤ .01, ***p ≤ .001, †*p* < .05. (Sig. results of LMM post-hoc contrasts predicting outcomes from time between T1 and T3, and T3 and T4). Note: IWS (internalized weight stigma).

### Exploring change in IWS (Hypothesis 1) and intuitive eating (Hypothesis 2) over time

To probe temporality of significant changes in IWS and intuitive eating across time within the linear mixed model (see *Data Analysis* section), we contrasted pre-treatment (T1) scores with post-treatment (T3) scores, and maintenance of change by contrasting T3 and follow-up (T4) scores.

Detailed results including effects of time and corresponding contrasts are shown in Table 2.

**Table 2.** Results of Linear Mixed Models predicting constructs by time, covarying for age, gender, and site, reporting unstandardized betas (B) and standard errors (SE).

Outcome	Effect	T1 - T3 contrast			T3 - T4 contrast		
		B	SE	*t*-test	B	SE	*t*-test
IWS (Time)	*F*(3,126) = 5.63***	−0.13	0.11	*t*(128) = −1.18	−0.32	0.12	*t*(124)=−2.67**
Age	*F*(1,69) = 0.23						
Gender	*F*(1,69) = 0.13						
Site	*F*(1,70) = 3.48†						
Intuitive eating (Time)	*F*(3,126) = 7.89***	0.13	0.05	*t*(127) = 2.50*	0.13	0.06	*t*(124) = 2.21*
Age	*F*(1,71) = 0.35						
Gender	*F*(1,72) = 0.09						
Site	*F*(1,72) = 1.52						

*p ≤ .025, **p ≤ .01, ***p ≤ .001, †*p* < .10. Note: IWS (Internalized Weight Stigma).

There were significant main effects of time across the four timepoints on increasing intuitive eating and decreased IWS (both *p *< .001). From T1 to T3, contrasts revealed increased intuitive eating (*p *= .01), and no significant change in IWS (*p* = .24). Maintenance contrasts from T3 to T4 revealed a further significant increase in intuitive eating (*p* = .03) and a significant decrease in IWS (*p* = .01). Study site had a marginal effect on IWS. On average across time, when compared to people at the urban site, those at the rural site reported marginally lower IWS (M. 2.66 ± 1.21 vs. 2.14 ± 1.13; B=0.53± 0.29, *t*(70)=1.87, *p* = .07).

### Exploring the association between change in IWS and intuitive eating (Hypothesis 3), and of each factor with change in mindfulness (Hypothesis 4a, 4b) and self-compassion (Hypothesis 5a, 5b)

All reported analyses utilize residualized change scores (see *Data Analyses* section). From pre- to post-treatment, decreased IWS was associated with increased intuitive eating, *r*(38) = -.43, *p* = .01. Increased mindfulness and self-compassion were both associated with increased intuitive eating, *r*(38) = .33, *p* = .04 and *r*(38) = .33, *p* = .04, respectively. Changes in mindfulness and self-compassion were unrelated with those in IWS, *r*(38) = -.14, *p* = .38 and *r*(38) = -.15, *p* = .36, respectively.

## Discussion

Internalized weight stigma (IWS) is increasingly implicated as a key contributor to poor mental, behavioral, and physical health, including eating disorders and poor cardiometabolic health, and approaches to mitigate its impact are sorely needed (Hunger, Dodd, & Smith, 2020; Pearl & Puhl, 2018; Schvey & White, 2015). Intuitive eating may prove a therapeutic target to reduce IWS (Clifford et al., 2015), and yoga may prove a novel vehicle for targeting improvement in both factors. Our study observed improvement in IWS and intuitive eating across the 16-week study timeframe among stressed adults reporting poor diet, with preliminary evidence that these changes were related over time. It is notable that these associations were observed in a yoga program that avoided any mention of diet, eating behaviors, weight stigma, body image, or weight loss/maintenance – consistent with a weight-inclusive (i.e. non-dieting, as opposed to weight-normative orweight loss focused) approach to health (Tylka et al., 2014).

Hypotheses 1 was primarily supported. IWS decreased, however, this change was only significant at follow-up (from T3 to T4), suggesting potentially delayed benefit of yoga participation. Intervention research suggests IWS can be challenging to change long-term (Pearl et al., 2020), and it is possible that more enduring changes in this construct related to yoga intervention accrue over a longer timeframe, and/or have delayed benefit. The yoga intervention assessed in the present study emphasized use of yoga postures, breathing exercises, and meditation ‘off the mat’ in stressful life situations, which may have accrued practice effects that took longer than the 12-week study duration to show. It is also possible that those who continued to practice yoga during the follow-up period were those who experienced delayed benefit. Future research would benefit from assessing continuation of yoga practice during the follow-up timeframe to assess whether it contributes to outcomes. Hypothesis 2 was also supported, suggesting yoga participation may foster intuitive eating among stressed adults with poor diet and that benefits may continue to accrue in the twelve weeks subsequent to treatment.

Reductions in IWS corresponded with increases in intuitive eating, supporting Hypothesis 3. Changes in IWS and intuitive eating over time may be reciprocal and/or interactive. While intuitive eating is clinically conceptualized as *protective* against the effects of IWS on behavioral health (e.g. Mathieu, 2009; Tribole & Resch, 2012), some evidence also suggests that high IWS may be a *risk* factor for low intuitive eating (Mensinger & Meadows, 2017; Webb & Hardin, 2015). Reductions in IWS may disrupt dichotomous cognitions and self-judgment related to maladaptive eating behaviors, food, and/or diet, thereby decreasing unhealthy dieting practices or disordered eating behaviors, and increasing intuitive eating. Alternately and complementarily, perhaps adoption of a more intuitive eating style mitigates self-stigma and self-criticism associated with IWS and the development of disordered eating behaviors. Future research would benefit from the use of designs and rigorous methodologies that afford prospective determination of temporality and causality between these factors within the natural environment such as Ecological Momentary Assessment, including among yoga practitioners to elucidate whether such changes are related to mindful yoga practices or non-specific factors.

Our finding that increased mindfulness and self-compassion associates with increased intuitive eating (supporting Hypotheses 4a, 5a) extends observational research findings linking these factors (Sairanen et al., 2015; Webb & Hardin, 2015). These results also offer preliminary support of Cox and Tylka’s (2020) theoretical model, which posits mindfulness and self-compassion are key contributors to positive embodiment, including the embodied practice of intuitive eating. Yet Hypotheses 4b and 5b were unsupported. Improvement in mindfulness and self-compassion did not correlate with reduced IWS, despite strong theoretical rationale and negative associations between self-compassion and IWS in prior cross-sectional research (Braun et al., 2021; Hilbert et al., 2015; Webb & Hardin, 2015). One explanation relates to our stressed sample, which excluded individuals with clinically elevated psychiatric symptoms, a history of psychopathology (including eating pathology), and those seeking to lose weight, all factors connected to elevated IWS (Pearl & Puhl, 2018). Mean levels of IWS in our sample were low relative to the cut point used to identify individuals with high IWS (2.7 vs. 4.0) (Pearl et al., 2020). Mindfulness and/or self-compassion may be most strongly linked to reduced IWS in samples that experience elevated IWS-related negative affect, such as individuals with eating pathology, or those who experience heightened weight-related discrimination and IWS (Braun et al., 2021a; Hilbert et al., 2015; Braun et al., 2016a). It also is possible that mindfulness and/or self-compassion may play an exogenous, mediating, or moderating role in the associations between intuitive eating and IWS, an interesting topic for future research. Better understanding how change in these factors may correspond with IWS and intuitive eating change during mindful yoga will inform future research, prevention, and intervention development efforts.

### Clinical implications

Although the present study cannot be generalized to clinical samples, clinicians and researchers who seek to examine yoga as an approach to reduce IWS among those with heightened levels, including people with eating disorders and poor cardiometabolic health (Hunger et al., 2020; Pearl & Puhl, 2018; Schvey & White, 2015), would benefit from carefully considering the approach and methodology (Cook-Cottone & Douglass, 2017). Emerging evidence implicates chronic discrimination – such as weight stigma – in the development of post-traumatic stress symptoms (Alessi, Martin, Gyamerah, & Meyer, 2013; Cheng & Mallinckrodt, 2015). Further, some research suggests certain forms of yoga may exacerbate, rather than alleviate, eating pathology (Cox, Ullrich-French, Cook-Cottone, Tylka, & Neumark-Sztainer, 2020; Domingues & Carmo, 2019, 2020), and care has been suggested to curate yoga spaces that facilitate positive embodiment (Cook-Cottone & Douglass, 2017). Therapeutic yoga is thus recommended that integrates a trauma-sensitive and identity-inclusive approach while emphasizing mindfulness and self-compassion, celebration of all body shapes and sizes, and inclusion of adaptations for all abilities (Cook-Cottone & Douglass, 2017; Emerson, 2015; Webb, Rogers, & Thomas, 2020). Additionally, due to elevated levels of shame and weight-related rejection sensitivity among people who experience weight stigma and heightened IWS (Hunger et al., 2020; Mensinger, Tylka, & Calamari, 2018), therapeutic yoga classes delivered in a virtual delivery format (i.e. telehealth) may yield initial enhanced acceptability and increase access and uptake.

An additional component of the broader system of yoga is the *sattvic yoga diet*, as articulated in yogic texts such as the Gheranda Samhitha (Desai, 1990; Saraswati, 2012). A sattvic yogic diet has been described as a ‘lacto-vegan nutritionally balanced, low fat, moderate protein, high complex carbohydrate diet’ that includes ‘fresh non-processed food with minimal non-irritating spices and condiments, and very easy to digest’ (Yogendra et al., 2004, Appendix 1). The intervention excluded the sattvic yogic diet given the parent study’s aim to determine whether integrated hatha yoga practices, excluding dietary recommendations, promote implicit improvement in diet, as well as the appropriate corresponding dose. Including a dietary change component would thus have confounded this primary outcome. Future research is warranted to clarify associations between a sattvic yogic diet and intuitive eating in the context of what has been termed "yoga bod" culture Webb et al., 2017), as well as eating pathology and orthorexia, in diverse populations.

### Limitations

Several limitations should be noted. The single-group research design does not allow for any causal inference of these results; it is unknown whether results represent effects related to the yoga intervention, regression to the mean, or non-specific factors. Results at best aid the generation of hypotheses for future research that should be examined in longitudinal and randomized controlled trials to elucidate temporality and causality. Moreover, because this study took place as part of a Kripalu yoga-based stress management intervention, it is unknown if these same relationships would exist in other yoga interventions which do not incorporate aspects of physical activity, positive embodiment, or mindfulness and self-compassion.

A related challenge in yoga research with multicomponent interventions is determining which specific factors contribute to beneficial change, given the widespread heterogeneity in such interventions. For instance, deep breathing, physical activity, mindfulness, and self-compassion alone are shown to contribute to many positive outcomes. Our parent clinical trial was designed as a first step towards understanding whether dietary change is observed during yoga, and the optimal ‘dose’ for such changes, rather than a test of which intervention components contributed to changes. Future research would benefit from careful consideration of appropriate attention- and contact-matched control groups to aid elucidation of unique and shared outcomes as well as pathways through which multicomponent yoga interventions may impact behavioral health. Approaches such as the Multiphase Optimization Strategy (MOST) that are designed to identify the specific and most efficacious intervention components would be ideal for this purpose (Collins, Murphy, Nair, & Strecher, 2005). Researchers may also consider use of the Essential Properties of Yoga Questionnaire (EPYQ), in development during this study, to characterize multicomponent yoga interventions and examine which components are most associated with beneficial change (Park et al., 2018).

Additionally, despite the use of diverse images in marketing materials, our sample was mostly White, non-Hispanic women with at least a college education who met extensive exclusion criteria, limiting external validity. These characteristics mirror much of yoga intervention and clinical trials research and warrant careful consideration in designing future recruitment plans (see Spadola et al., 2017, 2019; Webb et al., 2020 for promoting greater diversity in future yoga research). Relatedly, this study did not assess sexual orientation or gender identity, a regrettable limitation given health disparities, including elevated rates of eating disorders, among people who identify as sexual or gender minorities (Kamody, Grilo, & Udo, 2020; Valdiserri, Holtgrave, Poteat, & Beyrer, 2019).

Other limitations of the study include a high drop-out rate (which may indicate that our completers were unusually motivated), suggesting potentially biased results if these self-selected responders felt they learned more from the program than non-responders. Relatedly, the small sample size left the study underpowered to detect small effect sizes. While the LMM analyses were used in part to minimize the effect of missing data and optimize use of all available data, future work should rectify these gaps, including use of larger sample sizes to increase power. Last, our sample excluded all individuals with a history of eating pathology or actively seeking to lose weight, limiting generalization to these populations and underscoring the importance of future work in this area.

## Conclusion

Our pilot trial observed stressed adults with poor diet to report reduced internalized weight stigma (IWS) and increased intuitive eating following a yoga intervention, with these changes associated over time. Further, changes in mindfulness and self-compassion associated with those of intuitive eating. However, the sequence of temporality and causality of these changes remain unknown. While caution is warranted in interpretation of findings due to the single-group design, pending continued research, yoga may emerge a cost-effective tool to promote healthy eating behaviors and improve poor biobehavioral and metabolic health in high-stress populations at increased risk of chronic lifestyle diseases. More research, especially with diverse groups and using controlled designs, is warranted.

## Data Availability

The datasets generated during and/or analysed during the current study are available from the corresponding author on reasonable request.

## Clinical trials registry

The study protocol is registered in Clinicaltrials.gov (NCT02098018*).*
